# The Prospective Association Between Plasma Concentrations of Cellular Growth Factors and Risk of Heart Failure Mortality in Japanese Population

**DOI:** 10.2188/jea.JE20170123

**Published:** 2019-03-05

**Authors:** Ehab S Eshak, Koutatsu Maruyama, Hiroyasu Iso, Akiko Tamakoshi

**Affiliations:** 1 Public Health, Department of Social Medicine, Osaka University Graduate School of Medicine, Osaka, Japan; 2 Department of Public Health and Preventive Medicine, Faculty of Medicine, Minia University, Minia, Egypt; 3 Laboratory of Community Health and Nutrition, Special Course of Food and Health Science Department of Bioscience, Graduate School of Agriculture, Ehime University, Ehime, Japan; 4 Department of Public Health, Faculty of Medicine, Hokkaido University, Sapporo, Japan

**Keywords:** cellular growth factors, heart failure morality, nested-case control study, Japanese

## Abstract

**Background:**

Limited evidence is available on the association of insulin-like growth factors (IGFs) and risk of heart failure in population-based samples. We investigated whether serum IGFs concentrations can predict mortality from heart failure.

**Methods:**

We conducted a nested case-control study of 39,242 subjects aged 40–79 years who participated in the JACC study, a large Japanese prospective cohort study; participants provided serum samples and were followed up for 9 years. In heart failure cases and age-, sex-, community-, and year of blood withdrawal-matched controls, we measured serum concentrations of IGF-I, IGF-II, and IGF binding protein 3 (IGFBP3) and transforming growth factor (TGF-β1).

**Results:**

During the follow-up, there were 88 heart failure deaths (44 men and 44 women). Each increment of 1 standard deviation [SD] of IGF-II (120.0 ng/mL in women and 143.7 ng/mL in men) was associated with a 47% reduced risk of mortality from heart failure; multivariable odds ratio was 0.53 (95% confidence interval [CI], 0.30–0.94, *P*-trend = 0.03). The multivariable odds ratio in the highest quartile of IGFBP3 serum concentrations (≥3.29 µg/mL in women and ≥3.31 µg/mL in men) compared with the lowest (<2.11 µg/mL in women and <2.56 µg/mL in men) was 0.24 (95% CI, 0.05–1.11; *P*-trend = 0.12). No association was found between serum concentrations of IGF-I or TGF-β1 and risk of heart failure.

**Conclusions:**

Higher serum concentrations of IGF-II were associated with lower mortality from heart failure, which might suggest a possible role of IGF-II in the occurrence or prognosis of heart failure.

## INTRODUCTION

The failing heart results from a complex syndrome that based not only on the mechanical failure of the myocardium to provide adequate systematic perfusion, but also involves activation of various neurohumoral, immunologic, and biological mechanisms, as a result of or in accompany to myocardial injury.^1^ Provoking factors for this injury to the vascular smooth muscle cells include hypertension, diabetes, ischemic heart disease, congenital heart disease, cardiomyopathies, myocarditis, and other conditions.^1^

Insulin-like growth factors I and II (IGF-I and -II) are single-chain polypeptides, respectively composed of 70 and 67 amino acids, which are involved in proliferation, migration, contractility regulation, and apoptosis inhibition of vascular smooth muscle cells, like that of the myocardium.^2^^,^^3^ The concentrations of the circulating free IGFs are regulated by six IGF-binding proteins, largely IGFBP3, which not only regulates the active forms of IGFs but also modulates the binding of IGFs to their cellular receptors.^2^ Another growth factor peptide that involved in the vascular smooth muscle cells migration and proliferation is transforming growth factor β1 (TGF-β1), which can inhibit atherosclerosis.^4^

There has been growing evidence for the associations of IGFs concentrations with different provoking factors of heart failure; inverse associations of higher IGFs levels and positive associations of lower IGFs levels with risks of atherosclerosis,^5^ hypertension,^6^ diabetes,^7^ congenital heart disease,^8^ and cardiovascular disease,^9^^,^^10^ especially ischemic heart disease.^11^^,^^12^ Moreover, based on their promotive role in myocyte growth, high IGFs concentrations were found in patients with hypertrophic cardiomyopathies, both obstructive and non-obstructive types,^13^ but low concentrations were found in patients with heart failure.^14^^,^^15^ However, the evidence on prospective associations of the IGF-axis with heart failure is scarce and inconsistent; serum IGF-I concentrations were inversely associated with risk of incident heart failure in the Framingham Heart Study,^16^ but total IGF-I, IGFBP3, and insulin concentrations were not associated with risk of incident heart failure in the Cardiovascular Health Study.^17^ Moreover, no prior studies have examined the prospective associations between concentrations of IGF-II or TGF-β1 with risk of heart failure.

Therefore, within participants of the Japan Collaborative Cohort study (JACC), we investigated the associations of serum concentrations of IGF-I, IGF-II, IGFBP3, and TGF-β1 with mortality from heart failure in a nested case-control study.

## METHODS

### Survey population

In 1988–1990, the Ministry of Education and Science started to sponsor the JACC Study, which covered 110,585 inhabitants of 45 Japanese communities aged 40–79 years (64,190 women and 46,395 men). Self-administered questionnaires were distributed and participants were asked to report on their lifestyle, demographic characteristics and previous medical histories.^18^ Participants themselves or community representatives consented before enrolling in the study.

In a nested case-control study design, 35% of the residents (*n* = 39,242) consented to give blood samples.^19^ We excluded 916 women and 557 men with a history of cardiovascular disease, including myocardial infarction and/or stroke or a history of different types of cancers at baseline, which left 37,769 subjects (24,487 women and 13,282 men) to be enrolled in the current study. The human ethics review committees at Hokkaido and Osaka Universities have approved the study.

### Mortality surveillance

In this study, the follow up for mortality from heart failure ended by December 31, 1999. In each of the 45 communities, investigators conducted systematic reviews of the death certificates, which were sent to local community-public health center before centralization at the Ministry of Health and Welfare. For deaths that occurred from 1988–1994, codes of the 9th revision of International Classification of Diseases (ICD) was used to identify the death certificate-registered cause of death; ICD-10 codes were used for deaths from 1995–1997. Accordingly, ICD-9 code 402 and ICD-10t code 150 labelled heart failure deaths. The Family Registration Law of Japan, adhered to throughout Japan, requires registration of death. Therefore, it is believed that mortality surveillance is accurate. Censoring was applied for mortality cases that occurred after subjects shifted out from their original resident communities.

One control subject, free from any cardiovascular disease and matched for age (±5 years), sex, community, and year of blood sample withdrawal was randomly selected for each heart failure case.

### Determination of biochemical variables

Sera were prepared soon after blood sample reached the nearest laboratory to the surveyed district. Each participant’s serum sample was stored at −80°C until analysis in a single laboratory (SRL, Inc., Hachioji, Japan) after being divided over 3–5 tubes (300 µL per tube); none of the samples had been defrosted.^20^ Kits from R&D Systems (Minneapolis, MN, USA) were used in the sandwich enzyme-linked immunosorbent assay measurement of TGF-β1 concentrations; kits from Daiichi Radioisotope Laboratory (Tokyo, Japan) were used in the immuno-radiometric assay measurement of serum IGF-I, IGF-II, and IGFBP3 concentrations. Technicians who performed and interpreted all the assays were unaware whether the assayed sample was for a case or a control subject. Using various reference sera, the intra-assay coefficients of variation were 2.7–6.8% for TGF-β1, 2.2–3.5% for IGF-I, 2.7–4.5% IGF-II and 3.2–4.2% for IGFBP3 assays; respective inter-assay coefficients of variation were 4.2–6.2%, 1.1–4.2%, 4.2–5.5% and 5.3–8.8%.

An international member of the United States National Cholesterol Reference Method Laboratory Network (CRMLN), the Osaka Medical Centre for Health Science and Promotion, standardized the enzymatic methods of lipid measurements,^21^ which were done by an automatic analyzer (Hitachi 7600-210; Hitachi Medical Corp., Tokyo, Japan) at Kotobiken Medical Laboratories, Inc.

### Other covariates

The questionnaire asked the participants to report on their medical histories of chronic diseases, including liver and kidney diseases, stroke, myocardial infarction, different types of cancers, hypertension, diabetes, hypothyroidism, and other conditions via the following question: “Have you ever been diagnosed with any of the following?”, with possible answers of never, yes and on treatment, yes but already treated, and yes and not treated. Body mass index was measured from the self-reported height and weight as weight (kg)/height (m^2^). Smoking status was assessed by asking the participants to classify themselves as current smokers, ex-smokers, or never smokers, and current smokers were asked to report the number of daily smoked cigarettes. Similarly, the amount of daily ethanol intake among current drinkers of beer, wine, sake (Japanese rice wine), whisky, and shochu (Japanese spirits) were estimated in “go” units (conventional ethanol unit of sake) and transformed into grams by multiplying by 23 g/day. The blood pressure measurement was generally done twice in a seated position after 5 minutes rest via a mercury sphygmomanometer, and the representative values were used for the analyses.

### Statistical analysis

Mean values and proportions of baseline characteristics were measured and the statistical significance of the differences in these values among cases and controls were analyzed using the Student’s *t*-test and the χ^2^ tests, respectively. Also, in control subjects, differences in baseline risk characteristics were tested according to the sex-specific quartiles of cellular growth factors concentrations using analysis of covariance. The conditional logistic regression was used to compute the odds ratios (ORs) and 95% confidence intervals (CIs) of risk of heart failure mortality for each increment of one standard deviation [SD] of serum IGF-I, IGF-II, IGFBP3, and TGF-β1 concentrations and across biomarkers’ sex-specific quartiles, for which the cut-points were computed according to the concentrations in control subjects. The median value of the biomarkers’ concentrations in each biomarker’s categories was tested for linear trend using linear regression. Besides matching for age, sex, community, and year of blood sample withdrawal, the multivariate-adjusted model included sex-specific quartiles of serum total cholesterol concentrations (mmol/L), smoking status (non and current smokers), drinking status (non, former, and current ethanol drinker), systolic blood pressure levels (≤139, 140–159 and ≥160 mm Hg), antihypertension medication use (yes/no), sex-specific quartiles of body mass index (kg/m^2^), history of diabetes mellitus (yes/no), history of liver diseases (yes/no) and history of kidney disease (yes/no).

Statistical tests via SAS statistical package version 9.4 (Statistical Analysis System Inc., Cary, NC, USA) were conducted, with two-tailed *P*-values <0.05 considered statistically significant.

## RESULTS

Within the median 9-year follow-up, we ascertained 88 deaths from heart failure (44 in men and 44 in women). The mean age was 69.2 years for cases and 67.9 for controls, and there were no statistically significant differences between cases and controls in baseline characteristics, except for the levels of systolic blood pressure and antihypertension medication use, which were higher in heart failure cases than controls. Cases mean serum IGF-II and IGFBP3 concentrations were lower than those of controls (Table 1). Spearman correlation coefficients of IGF-II with IGF-I, IGFBP3, and TGF-β1 among controls were 0.36, 0.74, and 0.29, respectively, and those of IGFBP3 with IGF-I and TGF-β1 were 0.54 and 0.25, respectively (not shown in tables). Risk characteristics across controls’ sex-specific quartiles of cellular growth factors are shown in eTable 1.

**Table 1.** Age- and sex-adjusted mean values (standard deviation) and proportions of cardiovascular risk characteristics and selected biomarkers in cases and controls

	Heart failure		

	Cases	Controls	*P*-value^a^
Number of subjects	88	88	
Men, %	50	50	
Age, year	69.2 (7.4)	67.9 (6.6)	0.24
Body mass index, kg/m^2^	22.6 (3.5)	23.1 (3.0)	0.32
Systolic blood pressure, mm Hg	145.9 (21.0)	138.5 (19.1)	0.04
Diastolic blood pressure, mm Hg	83.7 (12.4)	80.8 (11.7)	0.17
Total cholesterol, mmol/L	5.2 (1.1)	5.1 (1.0)	0.54
Current smoker, %	32.5	20.7	0.09
Ethanol intake, g/day	32 (18)	27 (19)	0.38
Antihypertension medication use, %	27.6	14.8	0.05
History of diabetes mellitus, %	6.9	3.9	0.41
History of kidney diseases, %	5.8	5.4	0.99
History of liver diseases, %	4.4	2.7	0.67
IGF-I, ng/mL	107.2 (51.0)	110.2 (48.0)	0.69
IGF-II, ng/mL	532.4 (140.6)	588.3 (117.1)	0.01
IGFBP3, µg/mL	2.54 (0.81)	2.81 (0.67)	0.02
TGF-β1, ng/mL	33.92 (9.80)	36.01 (7.33)	0.11

^a^Calculated using Student’s *t*-test when comparing two means and using the χ^2^ test when comparing two proportions.

Table 2 presents age-, sex-, community-, and year of blood sample collection-matched and multivariable odds ratios for mortality from heart failure by 1-SD increment and across quartiles of serum IGF-I, IGF-II, IGFBP3, and TGF-β1 concentrations. In the age-, sex-, and community-matched model, higher serum concentrations of IGF-II and IGFBP3 tended to be inversely associated with mortality from heart failure in both quartile and continuous analyses. However, after adjusting for other covariates, the multivariable OR in the highest versus lowest quartiles was 0.46 (95% CI, 0.11–1.83; *P*-trend = 0.23) for IGF-II and 0.24 (95% CI, 0.05–1.11; *P*-trend = 0.13) for IGFBP3. The respective multivariable OR for 1-SD increment in IGF-II levels (120.0 ng/mL in women and 134.7 ng/mL in men) was 0.53 (95% CI, 0.30–0.94; *P*-trend = 0.03) and that for 1-SD increment in IGFBP3 levels (0.73 µg/mL in women and 0.78 µg/mL in men) was 0.69 (95% CI, 0.43–1.11; *P*-trend = 0.12). There was no association of IGF-I or TGF-β1 levels with mortality from heart failure in either continuous or categorical analyses.

**Table 2.** Conditional odds ratios (ORs) and 95% confidence intervals (CIs) for mortality from heart failure according to quartiles and 1-SD increment of selected biomarkers’ levels

	Quartiles of serum biomarkers concentrations^a^					OR per 1 SD^b^ increment	*P* value

	Q1 (Low)	Q2	Q3	Q4 (High)	*P*-trend		
**IGF-I**							
Number of cases	24	27	19	18			
Number of controls	22	21	23	22			
Age-, sex-, and community-matched OR	1.00	1.19 (0.50–2.79)	0.72 (0.30–1.75)	0.71 (0.28–1.78)	0.35	0.94 (0.66–1.33)	0.72
Multivariable OR^c^	1.00	1.34 (0.33–5.46)	0.78 (0.19–3.19)	0.59 (0.16–2.91)	0.47	0.95 (0.56–1.61)	0.84
**IGF-II**							
Number of cases	34	19	19	16			
Number of controls	23	20	23	22			
Age-, sex-, and community-matched OR	1.00	0.56 (0.23–1.39)	0.51 (0.22–1.17)	0.43 (0.17–1.06)	0.07	0.62 (0.44–0.89)	0.01
Multivariable OR^c^	1.00	0.85 (0.21–3.48)	0.53 (0.18–2.40)	0.46 (0.11–1.83)	0.23	0.53 (0.30–0.94)	0.03
**IGFBP3**							
Number of cases	38	13	25	12			
Number of controls	21	23	22	22			
Age-, sex-, and community-matched OR	1.00	0.31 (0.12–0.76)	0.63 (0.26–1.51)	0.31 (0.13–0.77)	0.02	0.70 (0.51–0.95)	0.02
Multivariable OR^c^	1.00	0.07 (0.01–0.39)	0.56 (0.13–2.49)	0.24 (0.05–1.11)	0.13	0.69 (0.43–1.11)	0.12
**TGF-β1**							
Number of cases	27	26	15	20			
Number of controls	22	22	23	21			
Age-, sex-, and community-matched OR	1.00	0.98 (0.43–2.22)	0.47 (0.19–1.20)	0.70 (0.29–1.69)	0.29	0.74 (0.53–1.05)	0.09
Multivariable OR^c^	1.00	2.90 (0.56–15.03)	0.37 (0.07–2.05)	2.30 (0.39–13.77)	0.53	0.94 (0.55–1.61)	0.82

SD, standard deviation.

^a^Ranges of IGF-I were <90 ng/mL for quartile 1, 90–120 ng/mL for quartile 2, 120–160 ng/mL for quartile 3, and ≥160 ng/mL for quartile 4 in men and <74, 74–110, 110–130, and ≥130 ng/mL in women. Respective ranges of IGF-II were <510, 510–580, 580–700, and ≥700 ng/mL in men and <500, 500–560, 560–650, and ≥650 ng/mL in women, those of IGFBP3 were <2.56, 2.56–2.93, 2.93–3.31, and ≥3.31 µg/mL in men and <2.11, 2.11–2.67, 2.67–3.29, and ≥3.29 µg/mL in women and those of TGF-β1 were <31.8, 31.8–38.5, 38.5–44.7, and ≥44.7 ng/mL in men and <29.6, 29.6–34.2, 34.2–39.2, and ≥39.2 ng/mL in women.

^b^1-SD IGFs and IGF-II were 57.6 ng/mL in men and 38.4 ng/mL in women for IGF-I and 143.7 ng/mL in men and 120.0 ng/mL in women for IGF-II, those of IGFBP3 were 0.78 µg/mL in men and 0.73 µg/mL in women and those for TGF-β1 were 8.0 ng/mL in men and 10.9 ng/mL in women.

^c^Multivariable OR: Further adjusted for body mass index (sex-specific quartiles), current smoking status (yes/no), alcohol consumption (current/ex-/non-drinker), systolic blood pressure levels (≤139, 140–159, and ≥160 mm Hg), antihypertensive medication use (yes/no), serum total cholesterol levels (sex-specific quartiles) and history of diabetes mellitus, kidney diseases and liver diseases (yes/no).

## DISCUSSION

This nested case-control study, conducted within a large prospective study, is the first to examine the prospective association of IGF concentrations with risk of mortality from heart failure and the first to show an inverse association between serum concentrations of IGF-II and risk of heart failure mortality. That inverse association remained after controlling for known heart failure risk factors. Reduced risk was observed with higher concentrations of IGFBP3, as well; however, the association did not reach statistical significance in the multivariable-adjusted model. Serum IGF-I and TGF-β1 concentrations were not associated with mortality from heart failure.

The biological mechanisms for the decreased risk of mortality from heart failure with higher concentrations of IGF biomarkers are not well-known; however, some suggestions are as follows: via endocrine, paracrine, and autocrine anabolic effects, the IGF-axis is involved in the cellular proliferation and growth of numerous tissues, including the heart. Higher levels of these biomarkers may help improve the cardiac development and hypertrophy and increase myocardial contractility.^3^^,^^4^^,^^8^^,^^13^^–^^17^ Low concentrations of these biomarkers were associated with almost all the provoking factors of heart failure: atherosclerosis,^5^ hypertension,^6^ diabetes,^7^ congenital heart disease,^8^ cardiovascular disease,^9^^,^^10^ ischemic heart disease,^11^^,^^12^ and cardiomyopathies.^13^

No previous studies have investigated the association between serum IGF-II concentrations and risk of heart failure, whereas the association between serum concentrations of IGF-I with risk of incident heart failure was examined in two previous studies and the results were inconsistent.^16^^,^^17^ Among 717 elderly participants in the Framingham Heart Study, 1-SD increment in log-transformed IGF-I concentrations (0.47 log-transformed IGF-I unit [log µg/L]) was associated with 27% lower risk of heart failure, and elders with serum IGF-I concentrations at or above the median level (140 µg/L), compared with those with serum IGF-I concentrations below the median, had 50% lower risk of incident heart failure.^16^ On the contrary, there was no association with serum concentrations of IGF-I among 566 incident congestive heart failure cases and 1,072 controls in a case-cohort study under the Cardiovascular Health Study.^17^ In the current study, we found no significant association between IGF-I concentrations and heart failure mortality.

The exact biological explanations of why serum concentrations of IGF-II, but not IGF-I, were associated with death from heart failure in our study are unclear. However, elevated concentrations of IGF-II receptor (a single-pass-transmembrane glycoprotein with higher affinity to bind to IGF-II than IGF-I), which mediates cellular entry and subsequent degradation of IGF-II,^22^ were shown to be involved in the pathology of end-stage heart failure via generating reactive oxygen species and promoting the cardiomyocyte apoptosis and also involved in the acute cellular rejection after heart transplantation via reacting as a receptor for granzyme B and inducing granzyme B-mediated necrosis of transplanted hearts.^23^ Thus, the observed association between IGF-II concentrations and heart failure death is plausible. Blood IGF-II levels remain stable lifelong; IGF-I levels decrease^2^^,^^25^ and the risk of heart failure increases with progressing in age.^24^ The mean age (78.4 years) of participants of the Framingham Heart Study, in which IGF-I was inversely associated with risk of heart failure,^16^ was 10 years higher than that of our participants (68.3 years). Accordingly, we assume that the inverse associations between higher serum concentrations of IGF-I and heart failure might be more evident in the elderly, where levels of IGF-I are low in general. However, there was no interaction with age for the association between IGF-I and risk of heart failure in our study participants.

The evidence on the association between IGFBP3 and risk of cardiovascular disease has been inconsistent; low IGFBP3 concentrations were associated with decreased risk of coronary heart disease^11^ in a Danish study, but associated with increased risk in a study of Americans.^12^ IGFBP3 not only works independently from IGFs’ effects via its direct action on cellular growth through its own receptors, but also it works in a way that extends the serum half-life of IGFs.^2^ In the current study participants, serum concentrations of IGFBP3 correlated with serum concentrations of IGF-II (*r* = 0.74), which might partially explain the observed insignificant reduced risk of heart failure with higher IGFBP3 concentrations.

Elevated plasma concentrations of TGF-β1 were found in heart failure patients.^26^ However, in the Cardiovascular Health Study, TGF-β1 concentrations were not associated with risk of heart failure.^27^ A recent review showed that there is a shortage of studies investigating the predictive value of TGF-β1 on mortality, and based on the available evidence, TGF-β1 was not one of the promising biomarkers for the prognosis of patients with heart failure.^28^

The strengths of the current study include the prospective design, the comprehensive cardiovascular surveys among a large population-based sample, the systematic surveillance of mortality and the acceptable reliability and validity for the measured biomarkers in a single laboratory. Still, there are some limitations: first, despite the high participation rate, only 35% of the total cohort participants were willing to give blood samples. However, we found no significant differences between participants who had given blood sample and those who had not regarding known mortality risk factors and participants’ characteristics.^19^ Therefore, the selection bias may be small for the evaluation of the associations between serum concentrations of the biomarkers and heart failure mortality. Second, frozen serum, which might have been subjected to changes due to long storage, was used to determine concentrations of the studied biomarkers. However, it was evident that the IGFs remained stable in serum samples collected for the JACC study participants and stored for 9 years at −80°C.^20^ Third, the death certificate diagnosis for heart failure was contaminated by deaths of unknown origin, especially cardiac arrest and arrhythmic deaths, because Japanese physicians tended to register mortalities from unknown origin and mortalities within end stages of chronic diseases as “unspecified heart failure” (I50.9 for ICD-10) before the introduction of the new form of death certificate in 1995, and the instructions by the Japanese Ministry of Health and Welfare to physicians to be careful of not using non-specific diagnoses of heart failure.^29^^–^^31^ Accordingly, more than one-third to half of heart failure deaths on death certificates were due to sudden death until 1994; however, after 1994 the proportion was reduced to approximately a quarter,^29^ and the heart failure mortality rate decreased by 70% from 1993–1995.^32^ On the other hand, the proportion of sudden death cases among heart failure deaths on the death certificate was reduced from 73.0% in 1994 to 34.2% in 1995–1996.^33^ These findings might indirectly imply that the validity of heart failure diagnosis in death certificate has been improved to some extent after 1994. Fourth, the number of cases across the quartiles of the studied biomarkers was small in the present study; thus, the low statistical power resulted in a sample size-variable OR in the multivariate model of the categorical analyses. Power calculations, which were based on 86–95 subjects due to the comparison only between the highest versus the lowest quartiles, showed that with the observed ORs for the highest versus lowest quartiles (0.59 for IGF-I, 0.53 for IGF-II, 0.24 for IGFBP3, and 2.30 for TGF-β1 and expected ratios of cases to controls of 0.47, 0.40, 0.37, and 0.46, respectively, for the reference groups, the design had a power of 22% for IGF-I, 40% for IGF-II, 79% for IGFBP3, and 8% for TGF-β1 for the detection of a difference with a significance level of 0.05). Last, it was shown that serum levels of IGF-I and IGFBP3 were significantly low in patients with hypothyroidism,^34^ a risk factors for heart failure.^35^ We did not add hypothyroidism in the multivariable-adjusted model because none of the cases or the controls in our study had reported having a history of hypothyroidism through the self-administered questionnaire.

In summary, higher IGF-II concentrations were inversely associated with risk of heart failure mortality, while the low mortality from heart failure with high IGFBP3 concentrations did not reach statistical significance. Serum concentrations of IGF-I and TGF-β1 were not associated with the risk in Japanese men and women. These findings suggest a potential contribution of serum concentrations of IGF-II to the development or prognosis of heart failure, and the measurement of this biomarker could help anticipate the risk of heart failure in subjects without cardiovascular diseases and cancer; however, further investigations are needed to confirm these associations.

## APPENDIX A. SUPPLEMENTARY DATA

eTable 1. Age- and sex-adjusted mean values and proportions of cardiovascular risk characteristics in control subjects according to quartiles of IGFs levels
