# Supplementary material for: The Prospective Association Between Plasma Concentrations of Cellular Growth Factors and Risk of Heart Failure Mortality in Japanese Population
Source: J Epidemiol. 2019 Mar 5;29(3):104–9. doi: 10.2188/jea.JE20170123 (PMC6375814; doi:10.2188/jea.JE20170123)
Supplement: Supplementary file 1 [file je-29-104-s001.pdf]

eTable 1. Age- and sex-adjusted mean values and proportions of cardiovascular risk characteristics in control subjects according to quartiles of IGFs levels

	**Quartiles of serum IGF-I**					**Quartiles of serum IGF-II**					**Quartiles of serum IGFBP3**					**Quartiles of serum TGF-β1**				
	**Q1(Low)**	**Q2**	**Q3**	**Q4 (High)**	***P*-trend**	**Q1(Low)**	**Q2**	**Q3**	**Q4 (High)**	***P*-trend**	**Q1(Low)**	**Q2**	**Q3**	**Q4 (High)**	***P*-trend**	**Q1(Low)**	**Q2**	**Q3**	**Q4 (High)**	***P*-trend**
Number of subjects	22	21	23	22		23	20	23	22		21	23	22	22		22	22	23	21	
Men, %	50	47.6	52.2	50	0.99	47.8	55	47.8	50	0.96	47.6	52.2	50	50	0.99	50	50	47.8	52.4	0.99
Age, year	68.2	69.8	68.3	65.4	0.13	70.1	69.1	66.6	65.9	0.02	72.3	67.2	65.3	67.0	<0.01	69.4	70.1	66.1	66.0	0.03
Total cholesterol, mmol/L	5.12	4.95	5.02	5.44	0.32	4.53	5.13	5.18	5.72	<0.01	5.01	4.91	5.28	5.34	0.18	4.77	5.28	5.24	5.25	0.10
Body mass index, kg/m^2	23.1	22.8	23.1	23.3	0.75	23.0	23.7	22.3	23.6	0.80	23.1	22.4	24.0	22.8	0.93	22.0	23.3	23.3	23.8	0.06
Current smoker, %	23.1	9.3	26.4	18.5	0.93	23.3	24.7	18.1	13.5	0.37	19.4	21.9	18.6	18.7	0.89	13.2	23.1	22.7	19.4	0.58
Ethanol intake, g/day	24.1	14.4	26.0	18.1	0.61	24.8	20.7	12.4	32.9	0.34	25.7	18.4	27.0	19.1	0.72	18.3	18.1	32.4	17.2	0.92
History of hypertension, %	28.0	14.6	36.3	38.2	0.32	37.3	17.1	31.4	31.5	0.97	22.6	31.8	24.7	37.9	0.39	23.5	27.6	47.8	19.3	0.92
History of diabetes mellitus, %	5.4	0.6	4.9	4.9	0.94	17.9	0.6	0.0	0.0	<0.01	1.7	13.7	0.0	0.0	0.31	14.8	0.6	0.0	0.0	<0.01
IGF-I, ng/ml	51.3	93.7	125.3	169.0	<0.01	91.1	107.3	111.6	131.3	<0.01	76.0	103.6	123.3	136.6	<0.01	108.3	109.8	105.6	117.6	0.62
IGF-II, ng/ml	525.0	595.1	590.6	642.7	<0.01	460.4	539.3	610.1	743.7	<0.01	502.5	530.5	613.5	705.4	<0.01	547.9	566.8	615.1	623.8	0.01
IGFBP3, μg/ml	2.3	2.8	2.9	3.3	<0.01	2.3	2.6	2.9	3.5	<0.01	2.0	2.5	3.0	3.7	<0.01	2.7	2.8	2.8	3.0	0.10
TGF-β	35.9	36.3	34.6	37.4	0.66	33.3	36.9	36.6	37.4	0.08	35.5	33.8	36.3	38.5	0.10	27.1	33.2	38.6	45.4	<0.01

IGF, insulin-like growth factor; TGF, transforming growth factor.

Ranges of IGF-I were <90 ng/mL for quartile 1, 90–120 ng/mL for quartile 2, 120–160 ng/mL for quartile 3 and ≥160 ng/mL for quartile 4 in men and <74 , 74–110, 110–130 and ≥130 ng/mL in women. Respective ranges of IGF-II were <510, 510–580, 580–700, ≥700 ng/mL in men and <500, 500–560, 560–650, and ≥650 ng/mL in women, those of IGF-BP3 were <2.56, 2.56–2.93, 2.93–3.31, and ≥3.31 μg/mL in men and <2.11, 2.11–2.67, 2.67–3.29, and ≥3.29 μg/mL in women and those of TGF-β1 were <31.8, 31.8–38.5, 38.5–44.7, and ≥44.7 ng/mL in men and <29.6, 29.6–34.2, 34.2–39.2, and ≥39.2 ng/mL in women.
